# Clinical characteristics and outcomes of patients with pediatric acute lymphoblastic leukemia after induction of chemotherapy: a pilot descriptive correlational study from Palestine

**DOI:** 10.1186/s13104-021-05678-6

**Published:** 2021-07-05

**Authors:** Ramzi Shawahna, Sultan Mosleh, Yahya Odeh, Rami Halawa, Majd Al-Ghoul

**Affiliations:** 1 Department of Physiology, Pharmacology and Toxicology, Faculty of Medicine and Health Sciences, An-Najah National University, New Campus, Building: 19, Office: 1340, P.O. Box 7, Nablus, Palestine; 2 An-Najah BioSciences Unit, Centre for Poisons Control, Chemical and Biological Analyses, An-Najah National University, Nablus, Palestine; 3 Department of Medicine, Faculty of Medicine and Health Sciences, An-Najah National University, Nablus, Palestine; 4 An-Najah National University Hospital, An-Najah National University, Nablus, Palestine

**Keywords:** Acute lymphoblastic leukemia, Induction chemotherapy, Treatment, Translocation, Palestine

## Abstract

**Objective:**

Pediatric acute lymphoblastic leukemia (ALL) is the most prevalent type of cancer among children. This study was conducted to describe and correlate the clinical characteristics and outcomes of treatment of patients with pediatric ALL in the main referral hospital in Palestine.

**Results:**

Complete data of 69 patients were included in this analysis. The majority (79.7%) of the patients had B-ALL phenotype. After induction chemotherapy, remission was experienced by the vast majority of the patients and 5 (7.2%) experienced relapses. Cytogenetics for patients with B-ALL phenotype indicated that 10 (18.2%) patients had t(12, 21) translocation, 5 (9.1%) had hyperdiploidy, 4 (7.3%) had t(1, 19) translocation, and 2 (3.6%) had t(9, 22) translocation. The initial white blood cells (p value < 0.001), absolute neutrophils (p value = 0.011), and hemoglobin (p value < 0.001) were significantly lower in patients with B-cell ALL. Platelet counts were significantly lower (p value = 0.012) in patients with splenomegaly and those with bleeding symptoms (p value = 0.008). Presence of palmar pollar was positively associated (p value = 0.035) with T-cell ALL. Presence of hepatomegaly was positively associated (p value < 0.001) with splenomegaly.

## Introduction

Cancer is the second most common cause of mortality among Palestinians in the West Bank and Gaza Strip [1]. In general, leukemias are the most common type of cancer among Palestinian children with an estimated incidence rate of 2.6 per 100,000 children [1, 2]. Acute lymphoblastic leukemia (ALL) is the most diagnosed tumor in pediatric population and the most frequent cause of death from cancer before the age of 20 [3]. According to some estimates, more than 75% of pediatric leukemias are ALL. On annual basis, about 6000 ALL cases are diagnosed in the US, of those, about 50% are children and teenagers [4, 5].

In Palestine, cancer care is highly fragmented and a high percentage of patients receive treatment outside the country [1, 6]. Currently, referral hospitals in which patients with pediatric ALL receive induction chemotherapy in Palestine are limited [1, 6]. Since its inception, An-Najah National Hospital has emerged as the main referral hospital for pediatric ALL in Palestine. Description of the epidemiological and clinical characteristics of patients with ALL in different nations has received considerable attention [7]. The Middle East Childhood Cancer Alliance (MECCA) collected clinical and demographic data on children with ALL from 16 countries in the Middle East [8]. However, Palestinians were not included in MECCA’s study. Currently, little is known on the epidemiological and clinical characteristics of patients with pediatric ALL among Palestinians [2]. Therefore, this pilot study was conducted to describe the clinical characteristics and outcomes of induction chemotherapy among patients with pediatric ALL in the main referral hospital in Palestine. The study also aimed to assess the associations between sociodemographic and clinical characteristics of the patients included in the study. The study provided insights into the clinical characteristics and outcomes of induction chemotherapy in Palestinian patients with pediatric ALL.

## Methods

### Study participants

Patients with pediatric ALL were included in this study. The inclusion criteria were: (a) patients who were 16 years old and younger, (b) admitted to the referral hospital with a diagnosis of pediatric ALL, and (c) received induction chemotherapy at the study site. With not restrict inclusion based on gender or admission dates. We excluded patients whose medical records were incomplete and those who received their induction chemotherapy outside the referral hospital. All patients with a diagnosis of pediatric ALL regardless of their gender or admission dates were screened against the inclusion and exclusion criteria.

### Study design, tools, and collection of data

This present study was a single-center, retrospective, observational study. The study used a descriptive correlational approach. In this study, paper-based and electronic medical records of patients with pediatric ALL were reviewed by field researchers. A data collection form that was created for this study using Excel Spreadsheets (Microsoft Excel, Microsoft Inc, US) was used to extract the pertinent data relevant to sociodemographic, physical examination, daily progress, hematological, clinical, bone marrow biopsies, flow cytometry, and cytogenetics. The data collected for this study were informed by previous studies [8–10]. Data were collected from the time the patient was admitted until outcomes of induction became available. The outcomes of the induction were assessed based on bone marrow biopsies performed on days 19–21 after initiation of induction chemotherapy. In this study, the outcomes were classified as follows: (a) remission (< 5% blast on bone marrow), (b) non-remission (≥ 5% blast on bone marrow), (c) relapse (≥ 20% blast in bone marrow, any blasts in CNS or both, or (d) death of the patient during the first admission for the induction chemotherapy.

### Statistical analysis

Data were entered into IBM SPSS for Windows v.21.0 (IBM Inc., Armonk, New York). Kolmogorov–Smirnov test was used to assess whether the data were normally distributed or not. Because the data were not normally distributed, the data were expressed using medians and their corresponding interquartile range (IQR). Categorical data were compared using Mann–Whitney *U* test. Correlation was investigated using Spearman’s rank correlation. Statistical significance was considered when the p value was < 0.05.

## Results

All medical records of patients who received induction chemotherapy at An-Najah National Hospital were reviewed. Complete records and laboratory reports were identified for a total of 69 patients. Patients with complete records and laboratory reports were included in the final analysis.

### Sociodemographic, clinical, and hematological variables of the study patients

Cytogenetic studies for the B-ALL phenotype showed that 10 (18.2%) patients had t(12, 21) translocation, 5 (9.1%) had hyperdiploidy, 4 (7.3%) had t(1, 19) translocation, and 2 (3.6%) had t(9, 22) translocation. Of all patients, 19 (34.5%) did not have any of the previously mentioned abnormal cytogenetics. Detailed sociodemographic, clinical, and hematological variables of the patients are shown in Table 1.

**Table 1:** Sociodemographic, clinical, and hematological variables of the patients with pediatric ALL included in the study (*n = *69)

Variable	n	%
Age (years)		
< 10	26	37.7
≥ 10	43	62.3
Gender		
Male	42	60.9
Female	27	39.1
Presence of abdominal pain		
Yes	22	31.9
No	47	68.1
Presence of fever		
Yes	37	53.6
No	32	46.4
Presence of bone pain		
Yes	30	43.5
No	39	56.5
CNS status^a^		
CNS 1	66	95.7
CNS 2	1	1.4
CNS 3	2	2.9
Presence of pallor		
Yes	50	72.5
No	19	27.5
Presence of anorexia		
Yes	9	13
No	60	87
Presence of hepatomegaly^b^		
Yes	34	49.3
No	35	50.7
Presence of splenomegaly^c^		
Yes	34	49.3
No	35	50.7
Presence of bleeding^d^		
Yes	15	21.7
No	54	78.3
Immunophenotype		
B-ALL	55	79.7
T-ALL	14	20.3
Cytogenetics for B-ALL		
t(12, 21)	10	18.2
Hyperdiploidy	5	9.1
t(1, 19)	4	7.3
t(9, 22)	2	3.6
Nil	19	34.5
Missing	15	27.3
Relapse^e^		
Yes	5	7.2
No	64	92.8
Remission^f^		
Yes	68	98.5
No	1	1.5
Hematological laboratory data at admission	Median	IQR
Hemoglobin (g/dL)	9.0	2.7
Platelet count (per mm^3^)	80000	157000
Initial WBCs count (cells/mm^3^)	7100	20600
Absolute neutrophils count (cells/mm^3^)	1420	2030

IQR interquartile range, WBCs white blood cells

^a^CNS1: WBC in CSF < 5 without WBC blasts, CNS2: WBC in CSF < 5 with WBC blasts, CNS3: WBC in CSF > 5 with WBC blasts

^b^Hepatomegaly: palpable liver below the costal margin

^c^Splenomegaly: palpable spleen

^d^Presence of bleeding symptoms like petechiae, epistaxis, bruising, gum bleeding, blood in stool

^e^Relapse: patient was in remission and developing any of the following: 1. More than 20% of blasts in bone marrow, or, 2. CNS status 2 or 3, or, 3. Both 1 + 2

^f^Remission: the percentage of blast cells in the bone marrow is less than 5% at the end of induction of chemotherapy

### Association between the different sociodemographic and clinical variables of the study patients with their hematologic laboratory findings

The median hemoglobin in patients who were 5 years of age and older (9.1 with an IQR of 2.5 g/dL) was significantly higher (p value = 0.007) than those who were younger than 5 years (7.8 with an IQR of 2.8 g/dL) (Table 2). The median platelet count in patients who did not have splenomegaly (122,000 with an IQR of 169,000) was significantly higher (p value = 0.012) than those who had splenomegaly (49,500 with an IQR of 145,850). The median platelet count for patients who did not have bleeding symptoms (103,000 with an IQR of 171,000) was significantly higher (p value = 0.008) than those who had bleeding symptoms (34,000 with an IQR of 66,050). The median hemoglobin for patients who had T-ALL (11.2 with an IQR 2.8) was significantly higher (p value < 0.001) than those who had B-ALL (8.4 with an IQR of 2.5). The median initial WBCs count for patients who had T-ALL (22,100 with an IQR 35,550) was significantly higher (p value < 0.001) than those who had B-ALL (5000 with an IQR of 10,950). The median absolute neutrophil count for patients who had T-ALL (2670 with an IQR of 4595) was significantly higher (p value = 0.011) than those who had B-ALL (1050 with an IQR 1730).

**Table 2:** Association between sociodemographic, clinical, and hematologic variables of patients with pediatric ALL

			Hemoglobin (g/dL)				Platelet count				Initial WBC count				Absolute neutrophil count			
Variable	n	%	Median	IQR	Mean rank	P value	Median	IQR	Mean rank	P value	Median	IQR	Mean rank	P value	Median	IQR	Mean rank	P value
Age (years)																		
< 10	26	37.7	7.8	2.8	26.6	0.007	57000	93000	30.2	0.119	7700	26400	37.3	0.454	1790	3830	35.0	0.289
≥ 10	43	62.3	9.1	2.5	40.1		118500	167000	37.9		4600	11028	33.6		1410	1640	30.0	
Gender																		
Male	42	60.9	8.5	3.0	38.3	0.088	87000	162500	36.1	0.563	7100	18700	38.4	0.077	1600	2700	34.9	0.137
Female	27	39.1	9.0	3.6	29.9		67000	171000	33.3		5000	7550	29.7		940	1870	27.9	
Presence of abdominal pain																		
Yes	22	31.9	9.0	3.5	34.2	0.822	88500	179000	35.6	0.867	5100	10670	36.4	0.690	1310	2450	31.9	0.982
No	47	68.1	8.8	3.0	35.4		64000	167000	34.7		7100	16700	34.3		1500	2390	32.0	
Presence of fever																		
Yes	37	53.6	9.0	2.6	37.8	0.217	78000	155250	34.7	0.909	4900	17178	34.8	0.942	1300	2595	34.8	0.188
No	32	46.4	8.5	2.5	31.8		64000	180500	35.3		7700	13950	35.2		1500	1759	28.7	
Presence of bone pain																		
Yes	30	43.5	9.1	2.9	34.8	0.928	59000	123000	33.2	0.506	5300	13100	33.4	0.553	1600	1800	34.4	0.341
No	39	56.5	8.7	2.2	35.2		88500	185500	36.4		5650	16725	36.3		975	3177	30.0	
Pallor																		
Yes	50	72.5	8.5	2.8	32.6	0.111	84000	157000	35.9	0.537	5300	13970	34.0	0.515	1420	2200	31.9	0.950
No	19	27.5	9.4	3.5	41.2		65500	211800	32.6		7300	19750	37.6		1450	2335	32.3	
Presence of anorexia																		
Yes	9	13.0	8.3	2.6	30.3	0.449	121000	168000	37.3	0.708	17700	32350	40.8	0.354	1500	3630	38.2	0.276
No	60	87.0	9.0	2.5	35.7		71000	158000	34.7		5000	12643	34.1		1410	2348	31.0	
Presence of hepatomegaly																		
Yes	34	49.3	9.0	1.6	36.1	0.644	50000	144500	31.5	0.151	5000	15750	36.6	0.513	1400	3030	32.8	0.735
No	35	50.7	8.3	3.8	33.9		118500	173000	38.4		6350	14350	33.4		1460	2075	31.3	
Presence of splenomegaly																		
Yes	34	49.3	9.0	1.7	37.1	0.381	49500	145850	28.8	0.012	7200	24300	38.0	0.216	1600	4295	35.3	0.180
No	35	50.7	7.8	3.0	32.9		122000	169000	41.0		5200	9250	32.1		1060	1615	29.0	
Presence of bleeding																		
Yes	15	21.7	8.0	4.5	30.6	0.333	34000	66050	22.9	0.008	9600	12335	39.1	0.367	1640	1170	35.2	0.481
No	54	78.3	8.9	2.2	36.2		103000	171000	38.4		4900	15300	33.9		1300	2725	31.2	
Immunophenotype																		
B-cell ALL	55	79.7	8.4	2.5	30.0	≤0.001	72000	156000	34.3	0.546	5000	10950	30.6	≤0.001	1050	1730	29.4	0.011
T-cell ALL	14	20.3	11.2	2.8	54.6		97500	264600	37.9		22100	35550	52.3		2670	4595	45.6	
Relapse																		
Yes	5	7.2	7.8000	1.35	31.8	0.711	60500	166750	38.2	0.711	1290	12353	24.7	0.233	290	3348	19.8	0.167
No	64	92.8	9.0000	2.80	35.3		84000	166000	34.8		5600	14500	35.8		1500	2150	32.8	

### Correlation between sociodemographic and clinical characteristics of patients with pediatric ALL

There was a positive correlation (p value = 0.035) between the presence of pallor at presentation and T-ALL immunophenotype. Again, there was a positive correlation (p value < 0.001) between the presence of hepatomegaly and the presence of splenomegaly (Table 3).

**Table 3:** Correlation between sociodemographic and clinical variables of patients with pediatric ALL

Variable	Correlation	Presence of abdominal pain	Presence of fever	Presence of bone pain	CNS status	Presence of pallor	Presence of anorexia	Presence of hepatomegaly	Presence of splenomegaly	Presence of bleeding	Immunophenotype	Relapse
Presence of abdominal pain	Spearman’s rho	–	0.01	− 0.04	− 0.01	0.14	− 0.08	0.20	0.20	0.17	0.04	− 0.07
	p value		0.918	0.772	0.943	0.240	0.512	0.106	0.106	0.170	0.770	0.561
Presence of fever	Spearman’s rho	0.01	−	0.11	0.08	0.21	0.10	− 0.07	− 0.07	0.00	0.11	− 0.08
	p value	0.918		0.359	0.489	0.087	0.408	0.559	0.559	0.980	0.373	0.533
Presence of bone pain	Spearman’s rho	− 0.04	0.11	–	0.05	0.02	− 0.08	− 0.16	− 0.22	− 0.04	0.22	− 0.13
	p value	0.772	0.359		0.697	0.889	0.517	0.182	0.068	0.763	0.064	0.278
CNS status	Spearman’s rho	− 0.01	0.08	0.05	–	0.02	0.08	− 0.07	− 0.07	− 0.05	− 0.11	0.06
	p value	0.943	0.489	0.697		0.851	0.500	0.556	0.556	0.654	0.379	0.627
Presence of pallor	Spearman's rho	0.14	0.21	0.02	0.02	–	0.14	0.09	− 0.11	0.09	0.25	− 0.08
	p value	0.240	0.087	0.889	0.851		0.243	0.470	0.385	0.467	0.035	0.524
Presence of anorexia	Spearman’s rho	− 0.08	0.10	− 0.08	0.08	0.14	–	− 0.04	0.13	0.11	0.09	− 0.11
	p value	0.512	0.408	0.517	0.500	0.243		0.760	0.270	0.373	0.470	0.376
Presence of hepatomegaly	Spearman’s rho	0.20	− 0.07	− 0.16	− 0.07	0.09	− 0.04	–	0.77	0.04	0.06	0.17
	p value	0.106	0.559	0.182	0.556	0.470	0.760		<0.001	0.727	0.597	0.158
Presence of splenomegaly	Spearman’s rho	0.20	− 0.07	− 0.22	− 0.07	− 0.11	0.13	0.77	−	0.11	− 0.01	0.06
	p value	0.106	0.559	0.068	0.556	0.385	0.270	< 0.001		0.355	0.952	0.625
Presence of bleeding	Spearman’s rho	0.17	0.00	− 0.04	− 0.05	0.09	0.11	0.04	0.11	−	0.00	− 0.15
	p value	0.170	0.980	0.763	0.654	0.467	0.373	0.727	0.355		0.975	0.227
Immunophenotype	Spearman’s rho	0.04	0.11	0.22	− 0.11	0.25	0.09	0.06	− 0.01	0.00	–	0.00
	p value	0.770	0.373	0.064	0.379	0.035	0.470	0.597	0.952	0.975		0.987
Relapse	Spearman’s rho	− 0.07	− 0.08	− 0.13	0.06	− 0.08	− 0.11	0.17	0.06	− 0.15	0.00	–
	p value	0.561	0.533	0.278	0.627	0.524	0.376	0.158	0.625	0.227	0.987	

## Discussion

This is the first description of hematological laboratory findings, signs and symptoms at presentation, immunophenotype, cytogenetics, and outcomes of induction chemotherapy for patients with pediatric ALL who received induction chemotherapy at the main referral hospital for pediatric ALL patients in Palestine. The study also established associations between hematological laboratory findings, sociodemographic, and clinical variables of the patients.

At presentation, the median hemoglobin concentration was 9.0 with an IQR of 2.7 g/dL. Hemoglobin levels reported in this study were comparable to those reported in a previous larger study in the Middle East [8] and other regional studies in Jordan [11] and Brazil [12]. Hemoglobin levels differed significantly between children older and younger than 5 years and those who had T-ALL or B-ALL. Findings of this study were consistent with those reported in previous studies [13, 14]. The median platelet count was 80,000 with an IQR of 157000 platelets/mm^3^. In a previous larger study in the Middle East, the platelet count was 36,600 platelets/mm^3^ [8]. In this study, patients who had splenomegaly and those who had bleeding symptoms had significantly lower platelet count than patients without splenomegaly and bleeding symptoms. Platelet counts after induction chemotherapy could be useful in treatment stratification of patients with pediatric ALL [15, 16]. Platelet counts could also be used in predicting prognosis and response to chemotherapy [15, 17]. The median initial WBCs count was 7100 with an IQR of 20,600 cells/mm^3^. The median initial WBCs counts were significantly different between patients who had T-ALL and B-ALL. Similarly, the absolute neutrophils counts were also significantly different between patients who had T-ALL and B-ALL. Our findings were consistent with the reported complete blood count and clinical findings of patients with pediatric ALL [13].

Pediatric ALL has many clinical manifestations and patients usually were symptomatic at presentation. Usually, symptoms result from dysfunction of different blood cells. In this study, pallor (72.5%), fever (53.6%), bone pain (43.5%), abdominal pain (31.9%) and bleeding symptoms (21.7%) were the main manifestations at presentation. Our findings showed that pollar was significantly associated with T-ALL. Presence of hepatomegaly was significantly associated with splenomegaly. Manifestations reported in this study were consistent with those reported in larger studies in the Middle East where fever (75.5%), bone pain (39.6%), and bleeding symptoms (30.8%) were the most frequent manifestations of pediatric ALL [8]. In Saudi Arabia, patients presented with fever (70%), bleeding symptoms (18%), and bone pain (10%) [10]. In this study, splenomegaly was reported in 49.3% of the patients. Al-Mulla et al. reported that 60.8% of patients with pediatric ALL had splenomegaly [8]. Among Saudi Arabians with pediatric ALL, 42% of the patients had splenomegaly [10]. Hepatomegaly was present in 49.3% of the patients with pediatric ALL included in this study. Findings of this study were consistent with those reported in the region among Arab populations in the Middle East and Saudi Arabia as well as those reported among Brazilians [8, 10, 12].

Consistent with international and regional studies, 79.7% of the patients had B-ALL and 20.3% of the patients had T-ALL. Findings of this study mirrored those reported in international studies as well as those reported in the Middle East and the region. B-ALL was prevalent in 85% of Middle Eastern patients with ALL [8], 82% of Saudi Arabian patients [10], and 89.5% of Brazilian patients [12]. Findings of this study reported that the vast majority of the patients (95.7%) had CNS status 1. Previous studies have shown that CNS infiltration altered protein profiling of the CSF [18]. Our findings were consistent with those reported among Middle Eastern, Saudi Arabian, Moroccan, and Brazilian patients with pediatric ALL [8, 10, 12, 19].

Although cytogenetic studies were not conducted for all patients, the findings of this study were consistent with those reported for the hyperdiploidy, t(12, 21), t(1, 19), and t(9, 22) translocations among patients with pediatric ALL. For example, in a study conducted in King Hussein Center in Jordan reported that about 12% of the patients with pediatric ALL were positive for translocation (12, 21), 1.7% were positive for translocation (1, 19), and 7.4% were positive for translocation (9, 22) [11]. Among Middle Eastern patients with pediatric ALL, 5.1% had (9, 22) translocation [8].

After induction chemotherapy, the vast majority (98.5%) of the patients included in this study had complete remission. In a larger Middle Eastern study, the remission rate was as high as 96.6% [8]. In Jordan, the remission rate was 7% [11]. In this study, 7.2% of the patients who showed complete remission developed relapse. Relapse rates after complete remission showed variability in previously reported studies in Jordan (9%) and Pakistan (20%) [11, 20].

## Limitations

First, this study was based on data collected from the medical record of the patients. Although a data collection form was specifically designed for this study and field researchers had access to the records as many times as they needed, the data collected could be biased by incorrectly entered information in the medical records. Second, this was a single-center study. Although An-Najah National Hospital has emerged as the main referral center for patients with leukemia, including those with pediatric ALL in the West Bank and Gaza Strip, the inclusion of other centers could have permitted a complete description of patients with pediatric ALL in Palestine. Third, the sample size included in this study was relatively small. This could be attributed to the fact that care for patients with cancer is based on fragmentation and patients often receive healthcare outside Palestine.

## Data Availability

The datasets used and/or analyzed during the current study are available from the corresponding author on reasonable request.

## Abbreviations

ALL: Acute lymphoblastic leukemia
B-ALL: B-cell precursor immunophenotype-acute lymphoblastic leukemia
CNS: Central nervous system
IQR: Interquartile range
IRB: Institutional review board
MECCA: Middle east childhood cancer alliance
T-ALL: T-cell precursor immunophenotype-acute lymphoblastic leukemia
WBC: White blood cell
WHO: World health organization
